# miR-143 and miR-145 inhibit gastric cancer cell migration and metastasis by suppressing MYO6

**DOI:** 10.1038/cddis.2017.493

**Published:** 2017-10-12

**Authors:** Chao Lei, Feng Du, Lina Sun, Ting Li, Tingyu Li, Yali Min, Aiying Nie, Xin Wang, Lei Geng, Yuanyuan Lu, Xiaodi Zhao, Yongquan Shi, Daiming Fan

**Affiliations:** 1 State key Laboratory of Cancer Biology, National Clinical Research Center for Digestive Diseases and Xijing Hospital of Digestive Diseases, Fourth Military Medical University, Xi’an, China

## Abstract

Metastasis is a major clinical obstacle responsible for the high mortality and poor prognosis of gastric cancer (GC). MicroRNAs (miRNAs) are critical mediators of metastasis that act by modulating their target genes. In this study, we found that miR-143 and miR-145 act via a common target gene, *MYO6*, to regulate the epithelial–mesenchymal transition (EMT) and inhibit metastasis. We determined that miR-143 and miR-145 were downregulated in GC, and the ectopic expression of miR-143 and/or miR-145 inhibited GC cell migration and metastasis. Furthermore, MYO6 was identified as a direct common target of miR-143 and miR-145 and was elevated in GC. Silencing of MYO6 resulted in a metastasis-suppressive activity similar to that of miR-143 and miR-145, while restoring MYO6 attenuated the anti-metastatic or anti-EMT effects caused by miR-143 and miR-145. Clinically, an inverse correlation was observed between miR-143/145 levels and MYO6 levels in GC tissues, and either miR-143/145 downregulation or MYO6 upregulation was associated with more malignant phenotypes in patients with GC. In conclusion, miR-143 and miR-145 suppress GC cell migration and metastasis by inhibiting MYO6 expression and the EMT, which provides a novel mechanism and promising therapeutic target for the treatment of GC metastasis.

Gastric cancer (GC) is one of the most common malignancies worldwide^1^ and is the second leading cause of cancer-related death in China.^2^ The majority of GC deaths are due to metastasis that occurs through a multistep process termed the ′invasion-metastasis cascade. In this multistep process, a series of genetic and epigenetic alterations affecting essential processes, including the epithelial–mesenchymal transition (EMT), occurs.^3^ A hallmark of the EMT is the loss of homotypic adhesion and cell polarity and the acquisition of mesenchymal characteristics, which is finely orchestrated by various regulators, including transcription factors, long non-coding RNAs, and microRNAs (miRNAs).^4^. However, the underlying mechanisms of EMT-mediated metastasis remain unclear.

miRNAs are a class of critical small non-coding RNAs that post-transcriptionally regulate target mRNAs by binding to their 3′-untranslated regions (3-UTRs), leading to translational inhibition or degradation of mRNAs.^5^ miRNAs can facilitate carcinogenesis by inhibiting tumor suppressor genes, or they can suppress tumor development and progression through inhibiting oncogenes.^6^ Recently, miRNAs have been shown to impact tumor metastasis in multiple human malignancies. For example, the miR-200 family promotes metastatic colonization by directly targeting ZEB1 and ZEB2 in ovarian cancer.^7^ miRNA-362-5p promotes tumor metastasis by targeting CYLD in hepatocellular carcinoma,^8^ and miR-150 promotes metastasis in non-small cell lung cancer by targeting FOXO4.^9^ In particular, miRNAs can modulate tumor metastasis by regulating the EMT. For example, miR-203 suppresses the bone metastasis of prostate cancer via the inhibition of the pro-EMT gene ZEB2,^10^ and miR-483-5p promotes the EMT in conjunction with an increase in the invasive and metastatic properties of lung adenocarcinoma by targeting RhoGDI1 and ALCAM.^11^ However, in GC, the second most common type of cancer in Asia in terms of incidence and cancer mortality, the roles of miRNAs, especially EMT-regulated miRNAs, remain to be elucidated.

miR-143 and miR-145 have been characterized as tumor suppressors in colorectal,^12^ breast^13^ and bladder cancer,^14^ but they promote tumor angiogenesis in lung cancer,^15^ indicating that the biological function of miR-143 and miR-145 in different cancer types needs to be clarified. Although miR-143 or miR-145 has been reported to be involved in GC development,^16^ the potential role of miR-143 and miR-145 in tumor progression, particularly in GC metastasis, remains largely unknown. Moreover, given that miR-143 and miR-145 are co-expressed from a single miRNA cluster and, in some cases, work together, determining whether miR-143 and miR-145 can act cooperatively to regulate GC metastasis is vital. In this study, we investigated the biological roles of miR-143 and miR-145 in GC metastasis *in vitro* and *in vivo*. In addition, we identified MYO6 as an important common target gene of miR-143 and miR-145 and further demonstrated that the tumor-suppressive and EMT-inhibitory roles of miR-143 and miR-145 are mediated by MYO6. These results enhance our understanding of GC metastasis and provide a potential target for miRNA-directed diagnostics and treatments for metastatic GC.

## Results

### miR-143 and miR-145 are downregulated in GC cell lines and specimens

**Figure 1:**
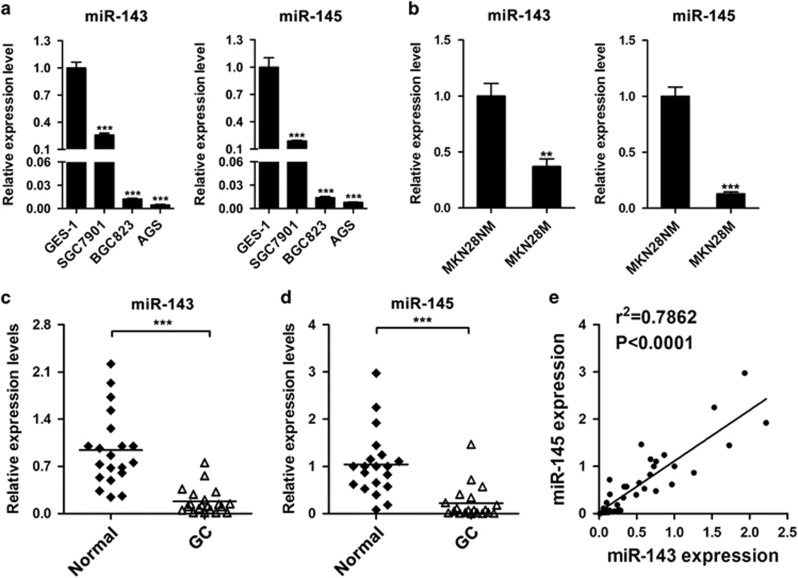
The expression levels of miR-143 and miR-145 in gastric cancer cells and clinical tissues. (**a**) The expression levels of miR-143 and miR-145 in three gastric cancer cell lines and one normal gastric epithelial cell line. (**b**) The expression levels of miR-143 and miR-145 in low and high-metastatic gastric cancer cell lines. (**c**,**d**) The relative expression levels of miR-143 and miR-145 in adjacent normal tissues and gastric cancer tissues. (**e**) Correlation between miR-143 expression and miR-145 expression in clinical samples. **P<0.01, ***P<0.001

To examine miR-143 and miR-145 expression during GC, we measured the levels of miR-143 and miR-145 in three GC cell lines, SGC7901, BGC823, and AGS, as well as the immortalized gastric epithelial cell line GES-1. We found that miR-143 and miR-145 expression was downregulated in SGC7901, BGC823, and AGS cells compared with that in GES-1 cells (Figure 1a). To investigate whether miR-143 or miR-145 is associated with GC metastasis, we further examined miR-143 and miR-145 expression in a pair of low- and high-metastatic GC cell lines, MKN28-NM and MKN28-M. miR-143 and miR-145 expression in the high-metastatic MKN28-M cells was markedly less than that in the low-metastatic counterpart MKN28-NM cells (Figure 1b). Moreover, we examined the expression levels of miR-143 and miR-145 in 20 pairs of GC tissues and their adjacent normal tissues and found that their levels were lower in GC tissues than in the adjacent normal tissues (Figures 1c and d). Intriguingly, a significant correlation between miR-143 and miR-145 expression was observed (Figure 1e). These data suggest that miR-143 and miR-145 might act as tumor suppressors and anti-metastatic factors in GC.

### miR-143 and miR-145 inhibit GC cell migration and metastasis

**Figure 2:**
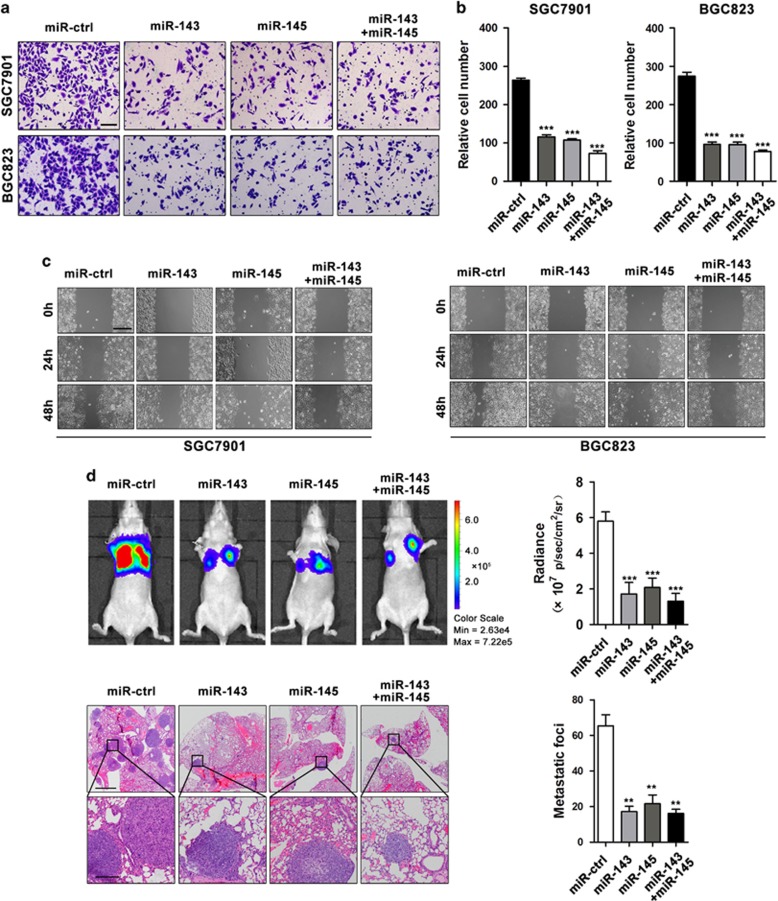
miR-143 and miR-145 inhibit GC cell migration and metastasis *in vitro* and *in vivo*. (**a**,**b**) Transwell assays of SGC7901 and BGC823 cells transfected with miR-143 and/or miR-145 mimics or miR-ctrl, scale bar: 100 *μ*m. (**c**) Wound-healing assays of SGC7901 and BGC823 cells transfected with miR-143 and/or miR-145 mimics or miR-ctrl, scale bar: 400 *μ*m. (**d**) Representative BLI images and H&E staining images of lung tissue sections from nude mice injected with BGC823 cells stably expressing miR-143 and/or miR-145, or miR-ctrl, scale bars: 500 *μ*m (top) and 200 *μ*m (bottom). **P<0.01, ***P<0.001

Previous studies have revealed that miR-143 and miR-145 inhibit GC cell proliferation and sensitize GC cells to cisplatin.^17, 18^ In this study, we mainly focused on the metastatic functions of miR-143 and miR-145 in GC. To this end, we performed transwell migration assays using SGC7901 and BGC823 cells transfected with either miR-143 or miR-145 mimics or both. miR-143 and miR-145 expression was confirmed by qRT-PCR after transfection (Supplementary Figure S1A). Overexpression of miR-143 and/or miR-145 inhibited GC cell migration (Figures 2a and b). Co-transfection of miR-143 and miR-145 resulted in a cooperative repression of cell migration that was greater than the effects of either miR-143 or miR-145 alone (Figure 2b). Consistently, a decrease in cell migration was also observed when miR-143 and/or miR-145 were upregulated in wound-healing assays (Figure 2c), suggesting that miR-143 and miR-145 can suppress migration *in vitro*. For *in vivo* experiments, BGC823 cells stably overexpressing miR-143 and/or miR-145 (Supplementary Figure S1B) were injected into nude mice through the tail vein. Five weeks after injection, bioluminescent imaging was performed to evaluate GC cell metastasis *in vivo*. We found that the bioluminescent intensity in the lung was reduced in the miR-143 and miR-145 groups compared with the intensity in the control group, and co-transfection of both miR-143 and miR-145 displayed a greater reduction in bioluminescent intensity compared with the effects of either miR-143 or miR-145 alone (Figure 2d). The mice were then killed, and metastatic nodules in the lungs were counted after H&E staining. We found that the number of metastatic nodules in the miR-143 and/or miR-145 groups was reduced compared with the number of nodules in the control group, and the number of metastatic nodules in the co-transfection group was even less than that in either the miR-143 or miR-145 group (Figure 2d). Taken together, these findings indicate that miR-143 and miR-145 can inhibit GC cell migration and metastasis in either an independent or cooperative manner.

### MYO6 is a direct common target of miR-143 and miR-145

**Figure 3:**
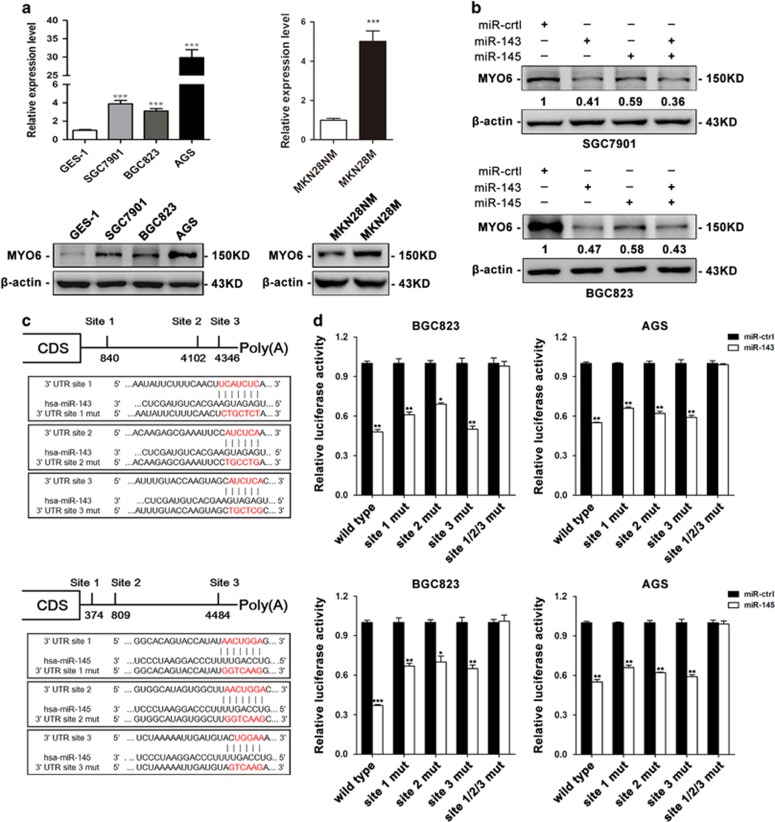
miR-143 and miR-145 inhibit MYO6 expression by targeting its 3′-UTR. (**a**) mRNA expression levels of *MYO6* (predicted target gene) in three gastric cancer cell lines, normal gastric epithelial cells and low and high-metastatic gastric cancer cell lines. (**b**) The protein expression level of MYO6 in SGC7901 and BGC823 cells after transfection with miR-143 and/or miR-145 mimics or miR-ctrl. (**c**) Diagram of the MYO6 3′-UTR-containing reporter construct. Mutations were generated at three predicted miR-143 and miR-145 binding sites located in the MYO6 3′-UTR. (**d**) Representative luciferase activity in BGC823 and AGS cells co-transfected with wild-type or mutated reporter plasmids and miR-ctrl, miR-143 or miR-145. *P<0.05, **P<0.01, ***P<0.001

To understand the mechanism underlying the metastatic-suppressive role of miR-143 and miR-145, we predicted potential target genes of miR-143 and miR-145 using integrated miRNA databases, including TargetScan, miRanda, PITA, DIANAmT, miRDB, miRWalk, RNAhybrid, PICTAR4, PICTAR5, and RNA22. From these prediction algorithms, eight candidates were identified as common target genes for miR-143 and miR-145 (Supplementary Figure S3). Among these genes, *MYO6*, which encodes an actin-based motor protein that is upregulated in various types of cancer,^19, 20, 21, 22, 23, 24^ has been widely reported to contribute to tumor cell migration and metastasis.^25, 26, 27, 28, 29, 30^ Therefore, we selected MYO6 for further investigation and measured its expression in GC cell lines at both the mRNA and protein levels. MYO6 expression was elevated in SGC7901, BGC823, and AGS cells compared with its expression in GES-1 cells (Figure 3a). In addition, MYO6 expression levels were higher in MKN28-M cells than in MKN28-NM cells (Figure 3a). To investigate whether miR-143 and miR-145 impact MYO6 expression, we examined MYO6 protein levels in SGC7901 and BGC823 cells transfected with miR-143 and miR-145 mimics and found that MYO6 was downregulated when either or both miRNAs were upregulated (Figure 3b). Furthermore, we employed a luciferase reporter system to confirm whether MYO6 was directly regulated by miR-143 and miR-145. The wild-type or mutant 3′-UTR of the MYO6 mRNA was inserted into a luciferase reporter vector (Figure 3c), and then each construct was co-transfected with miR-143 or miR-145 mimics in BGC823 and AGS cells. We observed that miR-143 and miR-145 inhibited the luciferase reporter activity of the wild-type MYO6 3′-UTR, but did not significantly change the luciferase reporter activity of the 3′-UTR with three mutated binding sites (Figure 3d). These results indicate that MYO6 is a direct common target of miR-143 and miR-145.

### Downregulation of MYO6 by miR-143 and miR-145 inhibits GC cell migration

**Figure 4:**
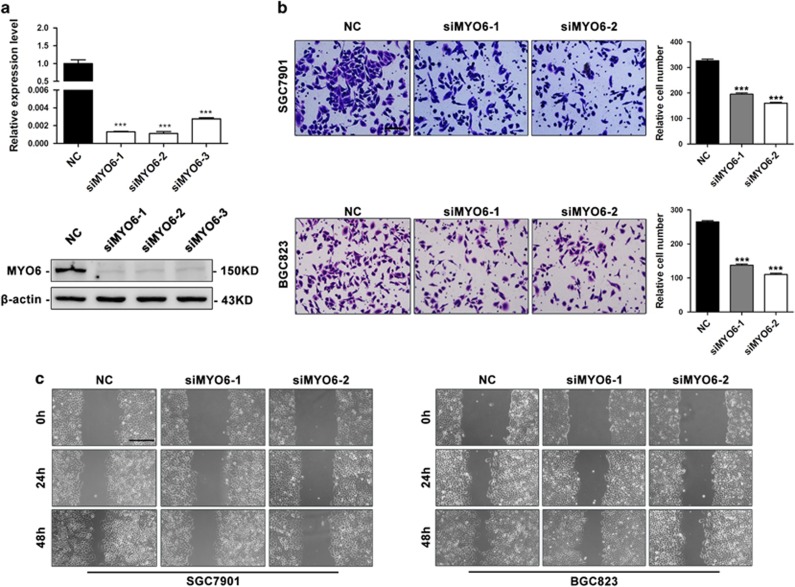
Downregulation of MYO6 inhibits GC cell migration and metastasis. (**a**) MYO6 mRNA and protein expression levels in BGC823 cells after transfection with MYO6 siRNA or negative control (NC). (**b**) Transwell assays in SGC7901 and BGC823 cells after transfection with MYO6 siRNA or NC, scale bar: 100 *μ*m. (**c**) Wound-healing assays in SGC7901 and BGC823 cells transfected with MYO6 siRNA or NC, scale bar: 400 *μ*m. ***P<0.001

**Figure 5:**
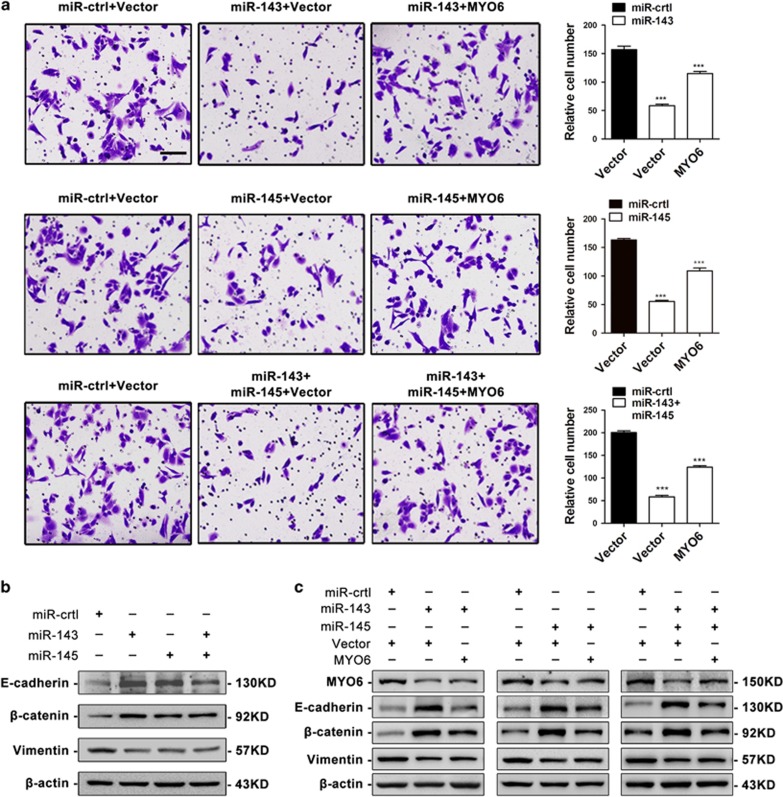
Restoration of MYO6 attenuates the suppressive effects of miR-143 and/or miR-145. (**a**) Transwell assays of BGC823 cells co-transfected with miR-143 and/or miR-145 mimics or miR-ctrl with either an empty vector or the MYO6 plasmid, scale bar: 100 *μ*m. (**b**) EMT marker protein expression in BGC823 cells transfected with miR-143 and/or miR-145 mimics or miR-ctrl. (**c**) EMT marker protein expression in BGC823 cells co-transfected with miR-143 and/or miR-145 mimics or miR-ctrl with either an empty vector or the MYO6 plasmid. ***P<0.001

MYO6 has been found to be elevated in and critical for maintaining the malignant properties of multiple types of human cancer.^19, 20, 21, 22, 23, 24, 25, 26, 27, 29, 31^ To examine whether MYO6 is involved in GC metastasis, we performed transwell migration assays and wound-healing assays in MYO6-silenced cells (Figure 4a). The downregulation of MYO6 decreased the migratory ability of SGC7901 and BGC823 cells (Figure 4b). Similarly, the silencing of MYO6 inhibited the wound-closing activity of SGC7901 and BGC823 cells compared with the controls (Figure 4c). To investigate whether MYO6 downregulation is essential for miR-143 or miR-145 to suppress migration, we co-transfected BGC823 cells with MYO6 or negative control vectors with either miR-143 or miR-145 mimics or both. As expected, restoration of MYO6 partially blocked the miR-143 and miR-145-mediated suppression of GC cell migration (Figure 5a). Taken together, these results demonstrate that MYO6 is an important functional target of both miR-143 and miR-145 and promotes GC migration.

### miR-143 and miR-145 inhibit the EMT by suppressing MYO6

Extensive studies have suggested that miR-143 or miR-145 regulate the EMT and EMT-related migration and metastasis.^32, 33, 34, 35, 36, 37^ Additionally, their common target MYO6 is also involved in EMT regulation.^31^ Therefore, we speculated that miR-143 and miR-145 might regulate the EMT through their shared target MYO6. To evaluate this possibility, we transfected BGC823 cells with miR-143 and miR-145 and then examined the expression of the epithelial markers E-cadherin and *β*-catenin, as well as the mesenchymal marker vimentin, using western blotting. We found that E-cadherin and *β*-catenin levels were increased but vimentin levels were reduced when miR-143 and/or miR-145 were overexpressed (Figure 5b). Furthermore, ectopic expression of MYO6 partially blocked the miR-143 and/or miR-145-mediated alteration of the EMT markers, with a reduction in E-cadherin and *β*-catenin expression and an increase in vimentin levels (Figure 5c). Consistently, immunofluorescent staining showed that miR-143 and/or miR-145 induced the expression of E-cadherin, but suppressed the expression of vimentin (Supplementary Figure S2A). We also observed that upregulation of miR-143 and/or miR-145 resulted in morphological changes from an extended morphology to more organized cell–cell contacts in BGC823 cells (Supplementary Figure S2A), but ectopic expression of MYO6 partially abolished the miR-143 and/or miR-145-mediated alterations in the EMT markers as well as the morphology changes (Supplementary Figure S2B). These results suggest that miR-143 and miR145 inhibit the EMT by regulating MYO6.

### MYO6 expression inversely correlates with miR-143 and miR-145 in GC tissues

**Figure 6:**
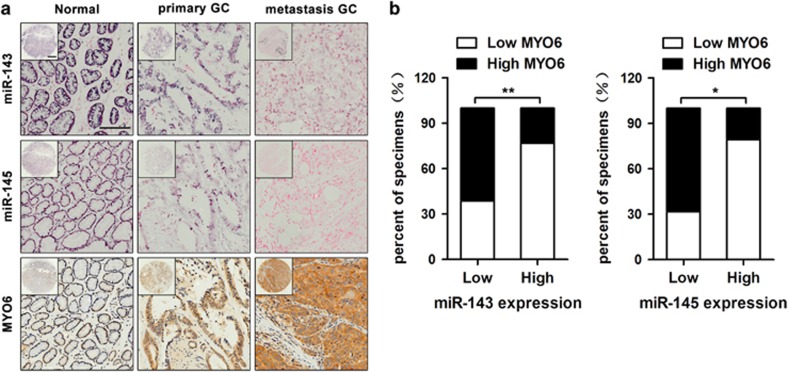
The expression levels of miR-143, miR-145 and MYO6 in GC specimens. (**a**) The expression levels of miR-143, miR-145 and MYO6 in normal (left), primary GC (middle) and metastatic GC (right) tissues, scale bars: 500 *μ*m (top) and 200 *μ*m (bottom). (**b**) The correlation between MYO6 expression and miR-143 and miR-145 levels in GC specimens. *P<0.05, **P<0.01

To examine the clinical relevance of miR-143/145 and MYO6, we measured the expression patterns of these factors using *in situ* hybridization and immunohistochemistry in commercialized tissue microarrays, which contain 24 normal tissues, 25 primary GC tissues and 21 lymphatic metastatic tissues. The *in situ* hybridization analysis revealed miR-143 and miR-145 expression in normal gastric tissue, but progressively less expression in primary GC tissues and metastatic GC tissues. In contrast, MYO6 expression increased gradually during GC progression as shown by immunohistochemical staining (Figure 6a). Likewise, an inverse correlation between miR-143/145 and MYO6 levels was observed in the statistical analyses (Figure 6b and Table 1). Furthermore, we performed correlation analyses and found that either downregulation of miR-143/miR-145 or upregulation of MYO6 was associated with larger tumor size and more frequent metastasis in GC patients (Table 2). These data suggest that miR-143/145 and MYO6 are inversely expressed, and their expression can be clinically correlated with more malignant GC phenotypes.

## Discussion

Previous studies revealed that miR-143 and miR-145 have dual functions and can suppress tumorigenesis in colorectal,^38^ breast,^13^ and prostate cancer^39^ but promote angiogenesis during lung cancer development.^15^ This inconsistency may be attributed to the expression or target gene differences of miR-143 and miR-145 in different cell types; therefore, investigating the functions of these miRNAs in diverse tumor cells is critical. In our study, we examined the roles of miR-143 and miR-145 in GC metastasis. We observed that miR-143 and miR-145 were downregulated in GC cells and tissues, and the difference was especially prominent when comparing their expression in high-metastatic GC cells and tissues with low-metastatic counterparts. Additionally, ectopic expression of miR-143 and miR-145 inhibited GC cell migration *in vitro* and metastasis *in vivo*. These findings demonstrate that miR-143 and miR-145 are important suppressors of GC metastasis. More recently, increasing evidence has indicated that miRNAs in the same cluster may possess related biological functions.^40, 41^ The miR-15a and miR-16-1 cluster targets CCND1 and WNT3A to inhibit cancer cell proliferation and invasion.^40^ The miR-106b-25 cluster activates TGF-beta signaling and induces the EMT via targeting SMAD7 in human breast cancer.^41^ Here, we examined the expressional and functional correlation between miR-143 and miR-145 and found the following: (1) miR-143 and miR-145 levels were positively correlated in 20 pairs of fresh GC samples, (2) miR-143 and miR-145 inhibited GC cell migration and metastasis individually or additively and (3) both miR-143 and miR-145 can directly target the 3′-UTR of their common target MYO6, which may serve as the molecular foundation of miR-143 and miR-145 cooperation. These findings provide novel evidence that individual miRNAs within a single cluster that are co-expressed but lack sequence homology can simultaneously and cooperatively regulate a common target.

MYO6, the only molecular motor protein that moves toward the minus ends of actin filaments,^19^ participates in the initiation and progression of a variety of tumors.^21, 22, 23, 24, 25, 26, 29, 30, 31^ MYO6 was found to be one of the genes most highly expressed in prostate cancers with more aggressive histological features,^21^ and MYO6 knockdown impairs the migration and soft-agar colony formation of prostate cancer cells.^30^ Similarly, MYO6 is abundantly expressed in high-grade ovarian carcinomas, and inhibition of MYO6 expression reduced tumor dissemination in nude mice.^19^ However, the role of MYO6 in GC metastasis was unclear. In this study, we investigated the roles of MYO6 and found that it functions as a metastasis-promoting oncogene, as it was upregulated in GC, and its silencing inhibited GC cell migration. Additionally, we identified the mechanism by which MYO6 dysregulation contributes to GC migration and metastasis. We found that the abnormal expression of MYO6 in GC was the result of miR-143 and miR-145 dysregulation, as supported by the following: (1) MYO6 was inversely expressed with miR-143 and miR-145 in GC, (2) miR-143 and miR-145 directly targeted MYO6 via binding to its 3′-UTR and (3) restoration of MYO6 canceled the migration-suppressive effects induced by miR-143 and/or miR-145. Our findings highlight a novel mechanism in which MYO6 acts as a functional target of miR-143 and miR-145 to regulate GC metastasis.

Accumulating evidence indicates that miRNAs are crucial regulators of EMT-mediated tumor metastasis. For instance, miR-203 targets ZEB2 and RUNX2 to suppress the EMT and bone metastasis of prostate cancer cells.^10^ miR-483-5p exerts pro-EMT and pro-metastatic functions by regulating RhoGDI1 and ALCAM in human lung adenocarcinoma.^11^ In GC, miR-338-3p inhibits EMTs by regulating ZEB2 and MACC1/Met/Akt signaling. miR-544a induces the EMT by activating the WNT signaling pathway.^42^ We previously found that miR-7 suppresses the EMT and GC metastasis by targeting IGF1R.^43^ In this study, we explored how miR-143 and miR-145 regulate the EMT in GC and found that the overexpression of miR-143 and/or miR-145 inhibited the EMT in GC cells, with a corresponding increase in E-cadherin and *β*-catenin levels and a decrease in vimentin expression. Furthermore, the ectopic expression of MYO6 rescued the EMT inhibited by miR-143 and miR-145, leading to reduced E-cadherin and *β*-catenin expression and an increase in vimentin levels in co-transfected cells. Our results demonstrate that miR-143 and miR-145 can inhibit the EMT in an MYO6-dependent manner, thus enhancing our understanding of miRNA-mediated EMT regulation.

In summary, we demonstrated that miR-143 and miR-145 were downregulated in GC cell lines and tissues. In addition, the overexpression of miR-143 and miR-145 inhibited GC metastasis *in vitro* and *in vivo* by targeting MYO6 and regulating the EMT process. The miR-143/145-MYO6 axis provides insight into the mechanisms underlying tumor metastasis and may serve as a novel therapeutic target for the treatment of metastatic GC.

## Materials and methods

### Cell culture

Human GC cell lines SGC7901, BGC823, AGS, and MKN28 and the immortalized gastric epithelial cell line GES-1 were purchased from the Cell Resource Center of the Chinese Academy of Sciences (Shanghai, China). The invasive cell subline MKN28-M and non-invasive cell subline MKN28-NM were created from the human GC cell MKN28 using the repeated transwell approach as previously described.^44^ Cells were maintained in Dulbecco’s modified Eagle’s medium (DMEM; Thermo Scientific HyClone, Beijing, China) supplemented with 10% fetal bovine serum (HyClone), 100 U/ml of penicillin, and 100 U/ml of streptomycin (HyClone) in a 37 °C humidified incubator with a mixture of 95% air and 5% CO_2_.

### Tissue collection

A total of 20 fresh primary GC samples and matched adjacent non-cancerous tissues were obtained from patients undergoing surgery at Xijing Hospital (Xi’an, China). All samples were confirmed by the Department of Pathology at Xijing Hospital and kept in a liquid nitrogen canister for further use. All patients provided informed consent for excess specimens to be used for research purposes. All protocols employed in this study were approved by the Medical Ethics Committee at Xijing Hospital.

### RNA extraction and real-time PCR

Total RNA from cell lines was extracted using an RNeasy Plus Universal Tissue Mini Kit (Qiagen, Hilden, Germany) as per the manufacturer’s instructions. miRNA from GC tissues was extracted using a miRNeasy Mini Kit (Qiagen). PCR primers for miR-143, miR-145, and U6 were purchased from RuiBoBio (Guangzhou, China). Primers for MYO6 and ACTIN were synthesized by TaKaRa (Dalian, China). The PCR primers for MYO6 were 5′-CAGAGCAACGTGCTCCAAAGTC’-3′ (Forward) and 5′-GAAGCGTTGCTG TCGGTTCA-3′ (Reverse). The primers for ACTIN were 5′-TCATGAAGTGTGA CGTTGACATCCGT-3′ (Forward) and 5′-CCTAGAAGCATTTGCGGTGCACG ATG-3′ (Reverse). cDNA was synthesized using a PrimeScript RT reagent kit (TaKaRa). Real-time PCR was performed using the SYBR premix Ex Taq II (TaKaRa). Fluorescence was measured in a LightCycler 480 system (Roche, Basel, Switzerland). The U6 small nuclear RNA and *β*-actin were used as internal controls for the miRNA and mRNA assays, respectively. Each sample was run in triplicate.

### miRNAs, siRNAs and plasmids

The human miR-143 mimics, miR-145 mimics and corresponding controls were synthesized by RuiBoBio (Guangzhou, China). Chemically modified small interfering RNAs were obtained from GenePharmagps (Shanghai, China). An MYO6 eukaryotic expression vector (MYO6/pcDNA3.1) was constructed by inserting the open reading frame of MYO6 into pcDNA3.1(+). For luciferase reporters, the 3′-UTR of MYO6 was first amplified from human genomic DNA, and then the miR-143 and miR-145 binding sites within the MYO6 3′-UTR were mutated to remove complementarity. Next, the mutant or wild-type 3′-UTR of MYO6 was cloned into the psiCHECK-2 luciferase vector for luciferase reporter assays. All plasmids were confirmed by sequencing.

### Cell transfection

For transfection, 100 nM miR-143 mimic, 100 nM miR-145 mimic, a mixture of each 50 nM miR-143 and miR-145 mimics, or 100 nM miR-ctrl was transfected into GC cells using the DharmaFECT Transfection reagent (Thermo-Fisher, Lafayette, CO, USA). The DharmaFECT Transfection reagent (Thermo-Fisher) was also used to transfect 100 nM siRNA into GC cells. The Attractene transfection reagent (Qiagen) was used for the transfection of 100 nM plasmid into GC cells. Transfections were performed following the manufacturer’s instructions. Forty-eight hours after transfection, cells were harvested for further investigation.

To generate stable cell lines, BGC823 cells were infected with miR-143 lentiviruses (multiplicity of infection, MOI=20), miR-145 lentiviruses (MOI=20), or the control vectors (MOI=20). The cells were selected with 2 *μ*g/ml puromycin for 2 weeks and then the infection efficiency was confirmed by qRT-PCR.

### Cell migration assays

For migration assays, transfected cells were resuspended in 200 *μ*l of serum-free DMEM medium and seeded onto Boyden chambers (Corning, NY, USA) with an 8.0-*μ*m pore membrane (5 × 10^4^ cells/well). The chambers were then incubated in DMEM with 20% FBS at 37 °C in 5% CO_2_. After 24 h, the cells adhering to the chamber’s lower surface were fixed, whereas cells remaining on the upper surface were removed. After staining in a dye solution containing 0.05% crystal violet, the cells from three randomly selected high power fields were counted under a microscope (Olympus, Tokyo, Japan). Migration assays were conducted in triplicate.

### Wound-healing assays

For wound-healing assays, transfected cells were plated in six-well plates. When the cell confluence reached 90–100%, a scratch was made using a 10-*μ*l pipette tip. The wounded monolayer was washed with PBS and imaged 0, 24, and 48 h after scratching using an Olympus camera system. Wound-healing assays were conducted in triplicate.

### *In vivo* metastasis assays

*In vivo* metastasis assays were performed as previously described. Briefly, BGC823 cells (1 × 10^6^ cells in 200 *μ*l of PBS) stably overexpressing miR-143, miR-145 or miR-ctrl were injected into nude mice (five in each group, female, nu/nu) through the tail vein. Five weeks after injection, the nude mice were injected intraperitoneally with D-Luciferin (150 mg/kg, Invitrogen, Carlsbad, CA, USA) in sterilized PBS, and bioluminescent images were captured using the IVIS Spectrum *in vivo* Imaging System (Perkin Elmer, Shanghai, China). Six weeks after injection, the mice were killed, and their lungs were dissected for H&E staining. The number of metastatic nodules was counted under a stereomicroscope (Olympus). All experimental animals were supplied by the Experimental Animal Center of the Fourth Military Medical University. All protocols for the animal studies were approved by the Fourth Military Medical University Animal Care Committee.

### Prediction of miR-143 and miR-145 target genes

We predicted potential direct common target genes of miR-143 and miR-145 using miRWalk (http://zmf.umm.uni-heidelberg.de/apps/zmf/mirwalk/index.html), an integrated miRNA database including TargetScan 5.1, miRanda, PITA, DIANAmT, miRDB, miRWalk, RNAhybrid, PICTAR4, PICTAR5, and RNA22. Genes predicted by at least seven algorithms to be common target genes of miR-143 and miR-145 were selected for further investigation.

### Protein extraction and western blotting

Whole-cell lysates were prepared in RIPA buffer (Beyotime, China). Primary antibodies against MYO6 (Cell Signaling Technology, Beverly, MA, USA), E-cadherin (Cell Signaling Technology), *β*-catenin (Cell Signaling Technology), vimentin (Cell Signaling Technology), and *β*-actin (Cell Signaling Technology) were used as per the manufacturer’s instructions. Densitometric analysis of specific blot bands was performed using ImageJ 1.48 software, and the intensity values were normalized against the b-actin loading control.

### Luciferase reporter assays

For the luciferase reporter assays, BGC823 and AGS cells were seeded onto 24-well plates and transiently transfected with an appropriate reporter plasmid and miRNA. Cells were collected and lysed for luciferase assays 48 h after transfection. Luciferase activity was measured using the Dual-Luciferase Reporter Assay System (Promega, Madison, WI, USA). Renilla luciferase was used for normalization. The transfection experiments were performed in triplicate for each plasmid construct.

### Immunofluorescence

Cells were plated onto glass coverslips, fixed with 4% paraformaldehyde for 10 min and permeabilized with 0.1% Triton X-100 in PBS for 15 min. Blocking solution was applied for 1 h at room temperature. Primary antibodies, mouse anti-human E-cadherin (Cell Signaling Technology), and rabbit anti-human vimentin (Cell Signaling Technology), were applied at 4 °C overnight. FITC-conjugated goat anti-rabbit and Cy3-conjugated goat anti-mouse secondary antibodies were loaded and incubated for 1 h at room temperature. Immunostaining signals and DAPI-stained nuclei were visualized at room temperature using a confocal microscope (FV10i; Olympus) equipped with a 10 × /0.30 NA objective lens (Olympus) and FluoView software (version 4.3; Olympus).

### Tissue microarray immunohistochemistry and *in situ* hybridization

GC tissue microarrays were purchased from Superchip (Shanghai, China). Each array contained adjacent gastric tissues, primary GC tissues and metastatic GC tissues from a total of 70 cases. *In situ* hybridization (ISH) was performed using miR-143 and miR-145 probes from Exiqon (miRCURY LNA detection probe 5′ and 3′-DIG labeled). The probes were detected using a digoxigenin antibody (Abcam, Cambridge, MA, USA), LSAB2 System-HRP (Dako Denmark A/S, Glostrup, Denmark) and the liquid DAB+ Substrate Chromogen System (Dako) following the manufacturer’s instructions. Immunohistochemical (IHC) staining was performed using an anti-MYO6 antibody (Abcam). The results of the IHC and ISH were scored independently by two pathologists in a blinded manner. The scoring was based on the intensity and extent of staining. Staining intensity was graded as follows: 0, negative staining; 1, weak staining; 2, moderate staining; and 3, strong staining. The proportion of stained cells per specimen was determined semi-quantitatively as follows: 0 for staining <1% 1 for 1–25% 2 for 26–50% 3 for 51–75% and 4 for >75% of the examined cells. The histological score (H-score) for each specimen was computed by the following formula: H-score=Proportion score × Intensity score. A total score of 0–12 was graded into negative (−, score: 0), weak (+, score: 1–4), moderate (++, score: 5–8) or strong (+++, score: 9–12). Samples with H-Scores >4 were determined to have high expression, and samples with H-Scores ⩽4 were determined to have low expression.

### Statistical analyses

SPSS software (version 19.0, SPSS Inc., Chicago, IL, USA) was used for statistical analyses. Continuous data are presented as the mean±S.E.M., and Student’s unpaired *t*-test was performed for comparisons between two groups. The linear correlation coefficient (Pearson’s *r*) was calculated to determine the correlation between miR-143 and miR-145 expression in clinical samples. Frequencies of categorical variables were compared using the *χ*^2^ test. *P*<0.05 was considered significant (*P<0.05, **P<0.01 and ***P<0.001).

## Publisher’s Note

Springer Nature remains neutral with regard to jurisdictional claims in published maps and institutional affiliations.

## Figures and Tables

**Table 1:** Expression of miR-143, miR-145 and MYO6 in normal, GC and metastatic tissues

**Gene**	**Tissue**	**Negative**	**Weak**	**Moderate**	**Strong**	***P*****-value**
*miR*-143	Normal (24)	2	6	11	5	0.040
	GC (25)	7	12	5	1	
	Metastasis (21)	4	13	2	2	
	Normal (24)	1	10	7	6	0.042
*miR*-145	GC(25)	1	11	9	4	
	Metastasis (21)	5	13	2	1	
	Normal (24)	3	19	1	1	0.048
*MYO*6	GC (25)	2	13	7	3	
	Metastasis (21)	0	10	6	5	

**Table 2:** Correlation of miR-143, miR-145 and MYO6 expression with patients’ clinicopathological variables in GC tissues

		**miR-143 expression**			**miR-145 expression**			**MYO6 expression**		
**Variables**	**Cases**	**Low**	**High**	***P*****-value**	**Low**	**High**	***P*****-value**	**Low**	**High**	***P*****-value**
*Gender*				0.468			0.659			0.596
Male	15	12	3		6	9		7	8	
Female	10	7	3		4	6		5	5	
										
*Age (year)*				0.645			0.223			0.450
⩽50	7	6	1		2	5		4	3	
>50	18	16	2		10	8		8	10	
										
*Tumor size (cm)*				0.011			0.042			0.036
⩽5	11	2	9		4	7		3	8	
>5	14	10	4		11	3		4	10	
										
*Metastasis*				0.016			0.021			0.033
No	9	2	7		2	7		7	2	
Yes	16	13	3		12	4		5	11	
