# Supplementary figures and images for: miR-143 and miR-145 inhibit gastric cancer cell migration and metastasis by suppressing MYO6
Source: Cell Death Dis. 2017 Oct 12;8(10):e3101–. doi: 10.1038/cddis.2017.493 (PMC5682659; doi:10.1038/cddis.2017.493)

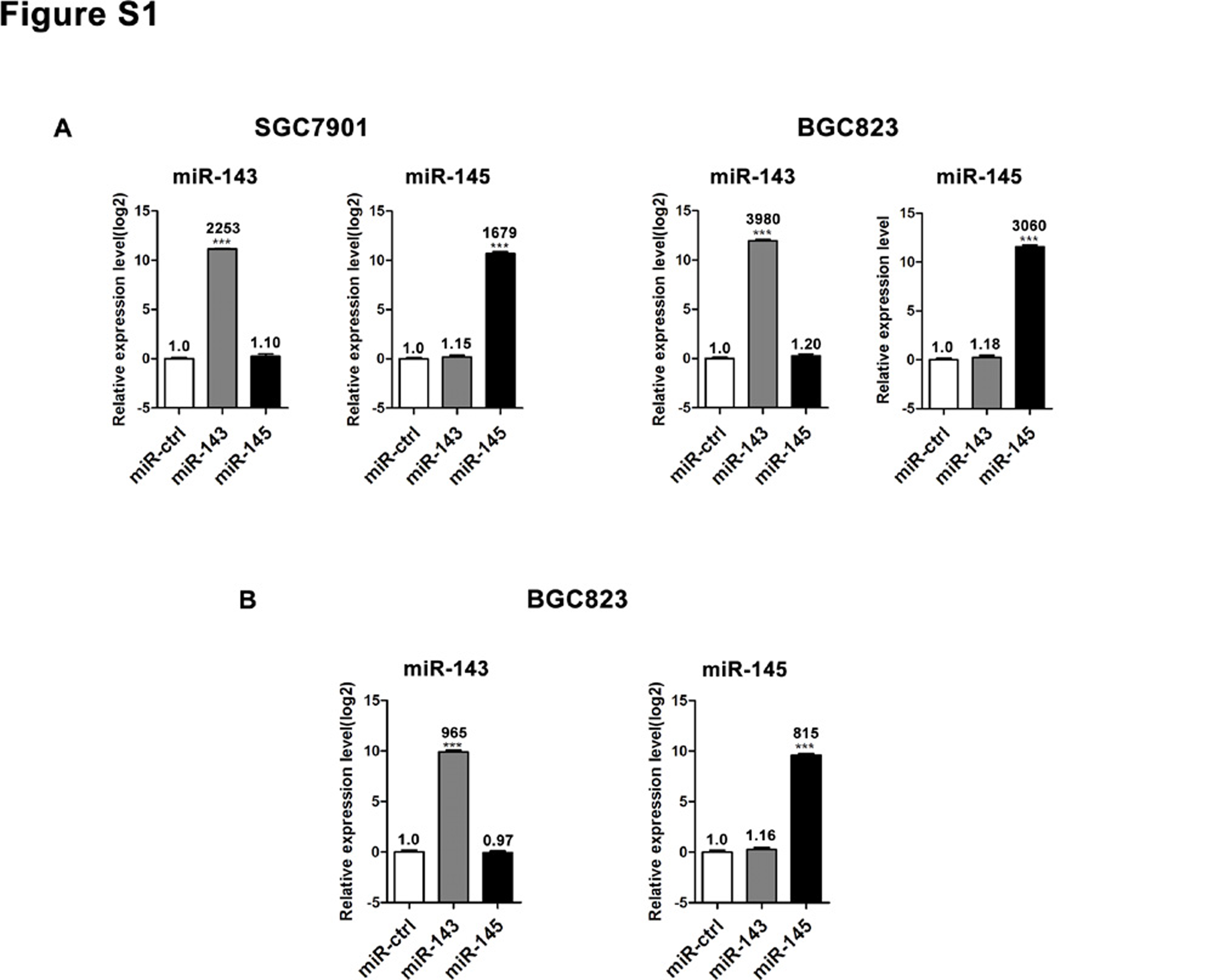

Supplement: Supplementary Figure 1 [file cddis2017493x1.tif]

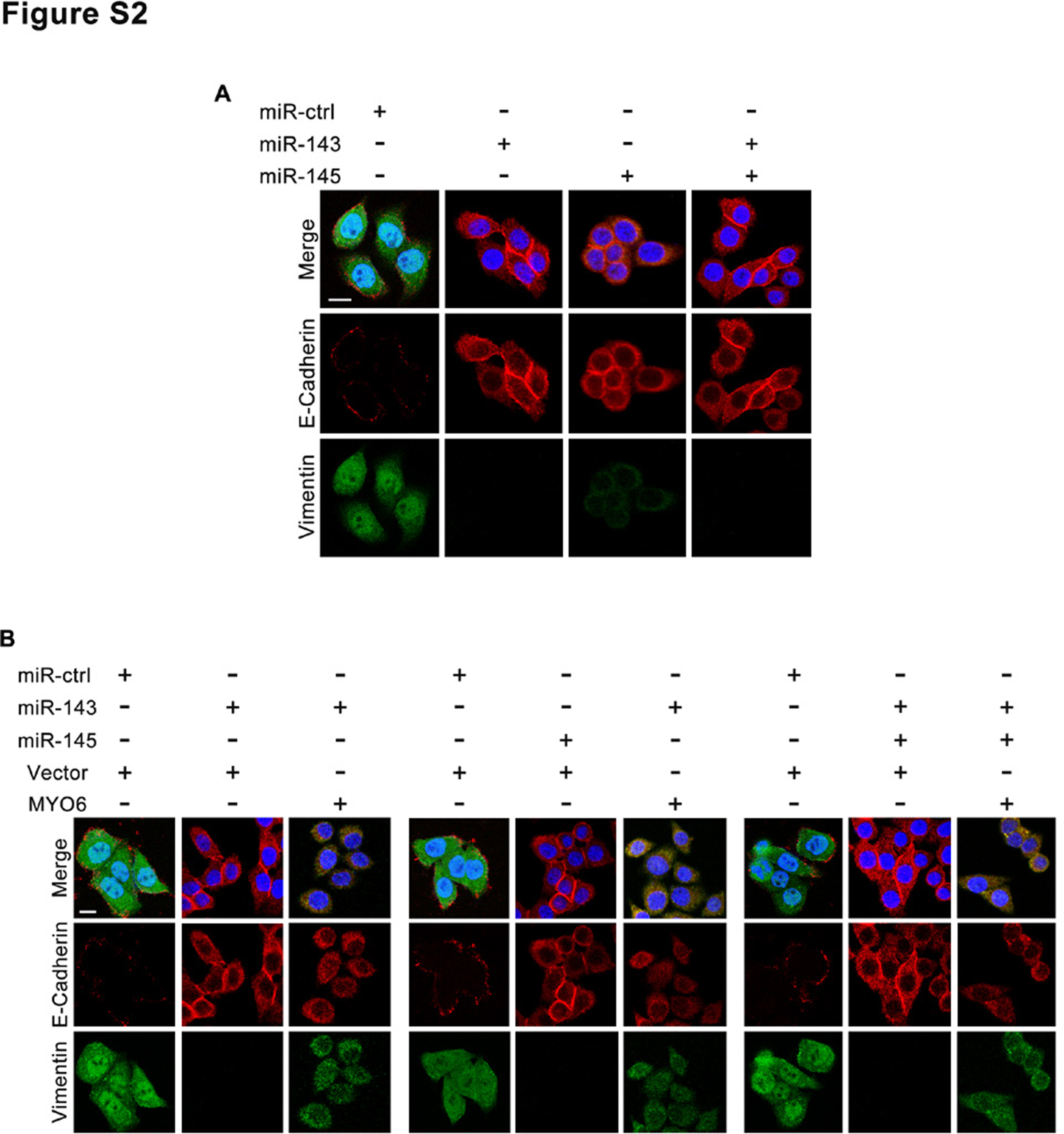

Supplement: Supplementary Figure 2 [file cddis2017493x2.tif]
