# Supplementary material for: miR-143 and miR-145 inhibit gastric cancer cell migration and metastasis by suppressing MYO6
Source: Cell Death Dis. 2017 Oct 12;8(10):e3101–. doi: 10.1038/cddis.2017.493 (PMC5682659; doi:10.1038/cddis.2017.493)
Supplement: Supplementary Figure Legends [file cddis2017493x3.doc]

**Supplemental Figure Legends**

**Figure S1. The transfection efficiency of miR-143 and miR-145 mimics in GC cells.**

**(A)** Real-time PCR analysis for miR-143 and miR-145 expression in SGC7901 and BGC823 cells after miR-143 mimics (miR-143), miR-145 mimics (miR-145), or miRNA mimics negative control (miR-ctrl) transfection. **(B)** Real-time PCR analysis for miR-143 and miR-145 expression in BGC823 cells infected with miR-143, miR-145, or miR-ctrl.

**Figure S2. The transfection efficiency of miR-143 and miR-145 mimics in GC cells.**

**(A)** Immunoﬂuorescence analysis of E-cadherin (red) and vimentin (green) in BGC823 cells transfected with the miR-143 and/or miR-145. **(B)** Immunoﬂuorescence analysis of E-cadherin (red) and vimentin (green) in BGC823 cells transfected with the miR-143 and/or miR-145, together with MYO6 plasmid. Merged pictures represent overlays of E-cadherin (red), vimentin (green) and nuclear staining by 4’,6-diamidino-2-phenylindole (DAPI; blue).
